# Cervical plate fracture: a rare complication

**DOI:** 10.11604/pamj.2015.20.266.5818

**Published:** 2015-03-19

**Authors:** Citisli Veli, Ibrahimoglu Muhammet, Civlan Serkan, Kocaoglu Murat

**Affiliations:** 1 Pamukkale University Medical Faculty, Department‘- of Neurosugery, Denizli, Turkiye

**Keywords:** Cervical plate, instrumentation, fracture

## Abstract

In traumatic and degenerative diseases cervical fusion with anterior cervical plate are commonly used. The increase in the use of cervical plate segment level is also increased risk of developing complications. This case report shows that the increase in the use of cervical plate segment level and also the complications in cervical spinal instrumentation, short-segment cervical plate rare case reported to be broken.

## Introduction

Cervical fusion with anterior cervical plate in traumatic and degenerative diseases is commonly used [1]. This article discusses the complications in cervical spinal instrumentation and short-segment cervical plate rare cases have been reported to be broken.

## Patient and observation

**Figure 1:**
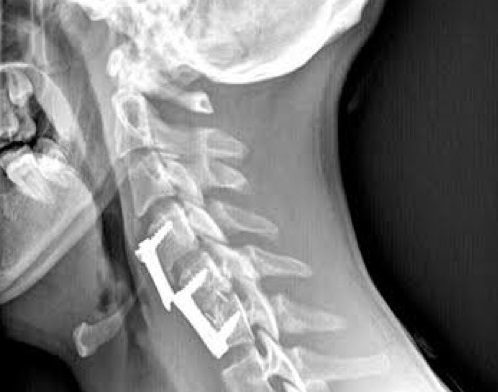
The plate is broken at the level of the anterior disc space C45 is seen

After a motor vehicle accident 18 years old male patient was brought to the emergency department. In neurologic examination; confused of consciousness, left hemiparesis, orientation and cooperation of the examinations in patients with limited, and upon detection level of C5-6 dislocation of grade 2, and patients were included in our service. In addition, patients who had pneumothorax, chest tube was inserted by chest surgery and the patient was operated. The disc of C56 space was determined by microscope and C5-6 Anterior cervical discectomy initiative was made using a microscope. One to C56 PEEK cage was placed in the disc space, and then plates were placed on anterior C4-C5-C6. Postoperative patients, Glasgow Coma Scale (GCS) as e3m3v neurosurgical intensive care were included. Patients without post-operative problems, was discharged after 2 months. Postoperative patient made physical therapy neck movement for exersize. Therefore, two years after surgery on the occurrence of severe neck pain were admitted again. Anterior cervical plate is broken at the level of the C45 range was determined by x-ray (Figure 1).

## Discussion

There are still ongoing debates about the pathology of cervical trauma is stable or unstable and intervention which will be banned from the anterior or posterior. Both initiatives have the advantages and disadvantages [2]. In the treatment of traumatic anterior instability ensuring stability cervical plating is very helpful and it is a widespread method in recent years [3, 4]. The goal of treatment of cervical fractures and dislocations: spinal cord protection is to ensure that no damage or more. The resulting distorted spine fracture or dislocation region is rotated normal sequence for stabilization [5]. Plaque stabilization of the anterior cervical spine; provide stability in the patient fully, spinal deformities that may occur in the post-operative period and decreased inhibition of bone graft migration and in patients the early stages of recovery provides significant advantages [6] After post-treatment of anterior cervical plating stability the specific complications can be listed as screw breakage, migration, plaque fracture, migration, pseudarthrosis formation, esophageal injury [3, 4, 7, 8]. In our case, we determined the grade 2 dislocation, closed subarachnoid space, traumatic disc detection and compression of the medulla from the ventral anterior so we used anterior approach.

## Conclusion

After spinal instrumentation early or late in the several complications arise. Cervical plaque rupture is one of them, but in this case, as mentioned, albeit short segments can be broken plates. For this reason, patients should be informed about the stabilization of the neck in the postoperative period and demanding excessive neck movement can cause damage to the instrument should be warned.
